# HIV/AIDS among Inmates of and Releasees from US Correctional Facilities, 2006: Declining Share of Epidemic but Persistent Public Health Opportunity

**DOI:** 10.1371/journal.pone.0007558

**Published:** 2009-11-11

**Authors:** Anne C. Spaulding, Ryan M. Seals, Matthew J. Page, Amanda K. Brzozowski, William Rhodes, Theodore M. Hammett

**Affiliations:** 1 Rollins School of Public Health of Emory University, Atlanta, Georgia, United States of America; 2 Abt Associates Incorporated, Cambridge, Massachusetts, United States of America; 3 Abt Associates Incorporated, Hanoi, Vietnam; Duke University Medical Center, United States of America

## Abstract

Because certain groups at high risk for HIV/AIDS (human immunodeficiency virus/acquired immunodeficiency syndrome) come together in correctional facilities, seroprevalence was high early in the epidemic. The share of the HIV/AIDS epidemic borne by inmates of and persons released from jails and prisons in the United States (US) in 1997 was estimated in a previous paper. While the number of inmates and releasees has risen, their HIV seroprevalence rates have fallen. We sought to determine if the share of HIV/AIDS borne by inmates and releasees in the US decreased between 1997 and 2006. We created a new model of population flow in and out of correctional facilities to estimate the number of persons released in 1997 and 2006. In 1997, approximately one in five of all HIV-infected Americans was among the 7.3 million who left a correctional facility that year. Nine years later, only one in seven (14%) of infected Americans was among the 9.1 million leaving, a 29.3% decline in the share. For black and Hispanic males, two demographic groups with heightened incarceration rates, recently released inmates comprise roughly one in five of those groups' total HIV-infected persons, a figure similar to the proportion borne by the correctional population as a whole in 1997. Decreasing HIV seroprevalence among those admitted to jails and prisons, prolonged survival and aging of the US population with HIV/AIDS beyond the crime-prone years, and success with discharge planning programs targeting HIV-infected prisoners could explain the declining concentration of the epidemic among correctional populations. Meanwhile, the number of persons with HIV/AIDS leaving correctional facilities remains virtually identical. Jails and prisons continue to be potent targets for public health interventions. The fluid nature of incarcerated populations ensures that effective interventions will be felt not only in correctional facilities but also in communities to which releasees return.

## Introduction

An earlier paper [1] estimated the share of selected infectious diseases, including HIV/AIDS (human immunodeficiency virus/acquired immunodeficiency syndrome), borne by persons who spent at least part of the year in correctional facilities (CFs) in the United States (US) in 1997. That paper examined the percentage of individuals with a particular disease in the total US population who passed through a US CF in 1997. Multiplying prevalence by estimates of the size of the populations that moved through CFs yielded projections that, among all persons in the US with HIV/AIDS, between 20% and 26% had been incarcerated for at least part of that calendar year [1]. The results were widely disseminated; as of September 2009, according to www.Scholar.Google.com, the paper has been cited 242 times. The findings communicated the challenges presented by the disproportionately high levels of infectious diseases in correctional settings. Furthermore, they demonstrated the importance of improving the health of the community by treating jail and prison inmates, since virtually all of those incarcerated eventually return to the community. Using the correctional setting as a venue for diagnosing HIV disease could benefit those passing through CFs as well as those living in the communities to which they return [2]. Primary and secondary prevention to reduce the high proportion of HIV/AIDS borne by incarcerated persons could help reduce its overall prevalence in the US.

Are CFs still important targets for public health interventions? If the criminal justice population represents a smaller share of the US HIV epidemic, intervening in the CFs may have a smaller impact on the US population as a whole and may be less cost effective than in 1997. If the number of inmates infected is still high, and if there are individuals in the correctional population who have poor access to healthcare when at liberty, CFs may remain important settings for public health interventions. Development of public health interventions that can capitalize on even brief access to incarcerated individuals may provide additional justification for public health agencies to work among persons passing through CFs.

A number of trends in the past nine years influence the answer to these questions. While HIV seroprevalence among people in CFs has declined from 2.1% in 1997 [3] to 1.7% in 2006 [4], the number of inmates behind bars at midyear 2006 was 130% of the that in 1997 (an increase from 1,725,842 to 2,245,189 persons) [5], [6]. At the same time, the way in which HIV affects the correctional population has changed. Highly active antiretroviral therapy (HAART) has markedly improved treatment for HIV, resulting in prolonged survival among both infected inmates and those previously incarcerated [7]. Rapid diagnostic algorithms for HIV [8]–[11] and brief behavioral interventions for prevention and management of HIV [12]–[14] have proven effective. Given these trends and their important policy implications, our objective was to recalculate the share of HIV/AIDS borne by correctional populations in the US.

We developed new estimates of the size of the population flowing through CFs in one year and the number of releasees that same year. Our estimation methodology accounts for the fact that, because so many individuals pass through more than once, neither the reported number of CF intakes nor of releasees represents unique persons passing though a facility. Using these estimates of the number of persons ever incarcerated during a year and the number of releasees, we developed estimates for the proportion of HIV borne by the respective populations.

## Methods

For this update, we estimated the share of HIV/AIDS in incarcerated persons during 2006. Using data from the Bureau of Justice Statistics (BJS), we estimated the percentage of inmates afflicted with HIV/AIDS and applied these percentages to the number of persons passing through CFs in 2006. Dividing this period prevalence by the number of infected persons nationwide provided estimates of the proportion of the US epidemic of HIV/AIDS borne by inmates and releasees.

We used a period prevalence rather than point prevalence because a 1-d snapshot of the incarcerated population fails to describe adequately how many different people enter and exit CFs during any given year. Jails and, to a lesser extent, prisons are characterized by flux [15]. For example, the Atlanta City Detention Center turns over its population rapidly, with a mean length of stay of just 22.5 h (Sgt. Rafael Bryant, personal communication, December 30, 2008). Prison inmates have a mean length of stay of 3 y [15].

Figure S1 displays the relationships and movements between locations within the correctional system, with the upper half representing custody and the lower half representing freedom. A hypothetical inmate may commit a crime, be arrested, be booked into a jail, and enter a cell pre-adjudication (a), go to court, and be sentenced and transferred to prison (e). For most individuals, this would take place within one year. Our methodology strove to account for this course of events and would count this individual as one person admitted to the correctional system. If the individual were eventually released on parole (k) and went on to recidivate within one year, she or he would be represented by the dotted line (i). If an individual was released from jail, either on bond or after charges were dropped, and was subsequently re-incarcerated in the same year on new charges, she or he would begin the travel through the criminal justice system at point (a) again. Our methodology counted this individual as just one person involved in the criminal justice system in the given year. Any recidivism after one year would make her or him a unique admittee in the next year.

### Number of Correctional Facility Inmates and Releasees

Data on the size of the stock population (those in cells and not moving in or out during a given year), total admissions, and total releases in 2006 were taken from publications of the BJS and the National Corrections Reporting Program (NCRP, website at http://www.icpsr.umich.edu/NACJD/ncrp). Estimates of the size composition of the stock population were taken from data collected at mid-year (June 30) 2006. To estimate the total number of jail admissions, we took data from the 2006 Annual Survey of Jails (available on the NCRP website) and used the method of Sabol and Minton [16] to derive a figure for total admissions.

Other than an estimate published in one previous paper [1], we are aware of no nationally representative data on re-arrest rates in jail detainees published in the peer-reviewed literature. For this paper, the estimate of the number of unique persons represented by total jail admissions in a year was derived from several sources of information: two smaller jails in jurisdictions with combination jails and prisons; data from the 1989 BJS Census of Jails; a previously published estimate by two of our authors (WR, TMH) based on data from the Drug Use Forecasting System [1]; and communication with four very large jail systems, collectively representing 2.5% of all admissions nationwide, that have inmate data reporting systems able to provide information on how many unique persons were represented in the pool of all admissions for a given year (see Table 1). An important caveat is that the various sources provide counts of slightly different, but closely linked events—arrest, booking, admission into a jail cell. The mean value of the number of entrances into the pre-trial division of the criminal justice system per individual in each data set was used as our estimate of admissions per individual, because the data from several sources provided similar estimates. Dividing the number of total jail admissions in a year by the average number of jail admissions per individual provides us with an estimate of the number of individuals admitted to jail in a given year.

**Table 1:** Number of Individuals Represented in Total Admissions.

Data Source and Type of Entry	Jurisdiction(s) and System(s)	Year (s)	Yearly Entries per Detainee
**Enumeration, awaiting trial in jail-prison system**			
Admissions^a^	Alaska Department of Corrections	2000	1.53
Admissions^a^	Delaware Department of Corrections	Average: 2000, 2001	1.38
**National survey data**			
All types of prior admissions for past 12 mo, for sentenced jail admittees^a^	Census of Jails: Nationally representative sample of inmates in jails	1989	1.41
Arrests, Drug Use Forecasting Survey^b^	Newly arrested males entering jails	1995	1.38
**Enumeration of entire population entering large jails**			
Admissions^c^	Philadelphia Prison (jail for City of Philadelphia)	2003	1.34–1.35
Admissions^d^	New York City Department of Correction (jail)	2006	1.43–1.46
Intake Transactions^e^	Washington DC Jail	2007 and 2008	1.43 and 1.42
Bookings^f^	Los Angeles County Jail	2008	1.26

a Unpublished estimates given in personal communication, 19 August 2009, Allen Beck.

b See Reference [1].

c Numerator: R. Eskind, personal communication, August 19, 2009, Philadelphia Prison Service, 2003–admissions, with range of admissions 32,573 by admission and discharge sheets, 32,614 by current data management system. Denominator of 24,290: J. Draine, July, 2009. Mental illness and Substance Abuse in Explaining Urban Jail Admission and Length of Stay. 31st Congress of the International Academy of Law and Mental Health. New York, NY.

d W. Franklin, personal communication, August 18, 2009. Data are from Population Research Unit, New York City Department of Correction. There were 105, 978 admissions in 2006, attributable to 72,457 individuals. There are 1,745 “known unknown” persons, namely intakes whose identity was not able, for whatever reason, to be linked to a NYSID or New York State fingerprint-based identification. Based on 72,457 known individuals, there may be up to 1,745 additional individuals represented.

e R. Chakraborty, personal communication, September 29, 2009., DC Department of Corrections. The ratio of Intake Transactions to Distinct Intakes was 1.43 in FY2007 and 1.42 in FY2008, according to FY 2007 and FY 2008 aggregate data, provided upon request. In FY 2007, there were 18,436 Intake Transactions and 12,910 Distinct Intakes.

f M. Malek and G. Cox, Los Angeles County Sheriff Department, personal communication, August 18, 2009. In 2008 the Los Angeles County Jail incarcerated 142,918 unique persons, who comprised 179,868 total bookings.

In order to determine the number of releases from jails in a 1-y period, we first calculated the net yearly growth of the jail system. The difference in average daily US jail population between 2005 and 2006 represents the balance between admissions and releases. The residual number of persons indicates the number of persons by which the jail population grew. By subtracting the amount by which the population grew from the total number of admitted individuals, we can estimate the number of unique releasees.

While jail admissions require an estimate based on national data and recidivism rates, prison records allowed us to perform a more complete enumeration of the number of individuals admitted and released in a year. In the current paper, we wanted to account for the growing number of persons admitted to state prison who are same-year recidivists, predominantly parole violators. In 2006, 17% of the state parolee population returned to incarceration [17]. This phenomenon is not as pronounced in the Federal Bureau of Prisons, where only 7% (8,521 of 128,774 parolees, or those on supervised release) returned to federal custody during the same year [17]. In the earlier paper, the number of prison releases was considered a reasonable estimate of the number of different people released from prisons because the average length of stay was greater than one year. Therefore, the estimate did not reflect the number of prisoners released on parole who returned to prison within a year ([i] in Figure S1) [1]. Information on the frequency of prison releasees who were readmitted and had more than one release in a single year were taken from NCRP. Because 2003 data were the latest available as of the preparation of this paper, we compared state-by-state data from 2002 and 2003 to assess stability and judge the suitability of extrapolation to 2006.

Records on all prison admissions from the 34 states reporting to NCRP were reviewed (according to date of birth, race, and state of imprisonment) to identify how many represented unique persons and how many prisoners were admitted multiple times. To extrapolate the figures for the remaining 16 states not included in NCRP data, we calculated the overall percentage of BJS-reported admissions comprising unique persons. We removed data from the single state with a proportion of unique releasees more than two standard deviations below the mean (California, with abnormally high recidivism) and from the one state with a proportion of unique releasees more than two standard deviations above the mean (North Carolina, with abnormally low recidivism) and recalculated the mean ratio of unique persons admitted relative to total number of admissions. We next examined the trend of state prison admission data from 2000 to 2006 as reported by BJS to yield a year-to-year growth factor that would allow us to extend 2003 data to 2006. BJS statistics on admissions to Federal prisons were added to yield the number of unique admittees to prisons nationwide. We used the same approach to derive unique releasees from prisons nationwide.

To estimate the total number of releasees in the community, we accounted for people who were released from jail into prison. Data from the State Court Processing Statistics of large urban counties (available on the NCRP website) provided an estimate of the proportion of individuals sentenced to prison who were detained pretrial. This group of individuals released from jail directly to prison was subtracted from the total number of jail releasees to obtain the number released to the community.

In order to compare our 2006 estimates to the 1997 estimates, we recalculated the 1997 prison release estimates using the new approach. Because a complete enumeration of unique jail releasees does not exist for 1997, we retained the earlier estimate of jail releasees.

### Determining Sex and Race/Ethnicity Proportions

We sought to describe the composition of incarcerated populations going in and out of CFs by race/ethnicity and sex. The total number of persons ever inside a CF and the total number of releasees is mostly influenced by the flow of persons through jails [1]. We weighted the cohort by sex and race/ethnicity proportionally to the demographics of its constituent parts. We relied on self-reports of sex and race/ethnicity in recent inmate surveys to derive demographic data. For prisoner race and ethnicity demographics, we used a published 2004 survey of state inmates [18]. For similar demographics for jail detainees, we used results from the BJS 2002 *Survey of Jail Inmates* available on the NCRP website. In both surveys, the term “whites” and “blacks” stood for non-Hispanic whites and blacks, respectively; “Hispanic” referred to those of Hispanic ethnicity regardless of race.

### Methods for Deriving HIV/AIDS Data

#### HIV in jail and prison populations

Data derived from BJS 2002 *Survey of Jail Inmates* were used to estimate overall HIV prevalence among jail detainees who had been tested for HIV [19]. Additional information was taken from CDC data on HIV prevalence among untested jail detainees [10].

BJS produces annual estimates of the number of HIV/AIDS cases in prisons based on reports from state and federal prison systems. Testing practices vary widely between states, with some systems testing all incoming inmates, some testing all outgoing inmates, others conducting routine seroprevalence surveys, and still others with little or no routine testing. The state with the highest prevalence of HIV, New York, uses an innovative methodology for estimating stock population prevalence; blinded seroprevalence surveillance has been conducted on a sample of sequential admissions every 2–3 y since 1988. Examining the percentage of the current population that entered during each 2- to 3-y entry period, one can assign an estimate of the HIV prevalence to the block and thus derive an HIV prevalence estimate for the stock population as a whole (methodology published in BJS bulletin [20] and L.N. Wright, personal communication, July 30, 2008).

The earlier paper estimated that the true range of HIV was between 1 and 1.5 times the BJS estimate [1]. For the present paper, we took the BJS estimates as given since no evidence has recently emerged that these estimates substantially deviate from true values. Rates for HIV prevalence by race and sex were taken from the 2004 State Inmate Survey. The inclusion criteria for falling into the category of “other,” a category which comprises those not fitting into three major race/ethnicity categories, either because of multiple races, membership in another racial group, or non-response, was not uniform and so minimum and maximum values based on extremes of possible classification schemes were estimated.

#### HIV in total population

In late 2008, the CDC released data for 2006 on HIV/AIDS prevalence by race and ethnicity in 33 states with name-based reporting, based on new methodology [21]. Data on AIDS prevalence is collected in all states and reported to the CDC [22]. We used these CDC data for estimates of AIDS prevalence in the total population.

### Sensitivity Analysis

We performed a bidirectional sensitivity analysis to assess the stability of our estimate of the proportion of HIV/AIDS borne by those released from CFs in 2006. To assess the extreme upper limit of our estimate, we assumed that all jail and prison admissions and releases reported by the BJS were, in fact, unique persons (inflating the numerator) and employed the lower bound of the CDC estimate for HIV/AIDS in the US (deflating the denominator). To obtain a lower limit, we assumed that the average jail inmate was incarcerated 1.5 times, and employed our point estimate for unique prison releases and the upper bound of the CDC estimate for HIV/AIDS in the US. We estimated how many fewer HIV-infected persons would be in the cohort of releasees if this higher return rate were used and assessed whether or not the number of releasees would decline by the same number of persons as did the number of admittees. To estimate the difference in the likely number of HIV-infected persons in this release cohort, we multiplied the HIV seroprevalence of jail detainees by the number of persons by which the release cohort declined.

## Results

### Static or “Stock” Population, June 30, 2006

Estimates for the “stock” population, the number of persons behind bars on June 30, 2006, are from the BJS, which estimated that 1,479,179 persons were incarcerated in prison that day. Adding to this the 766,010 jail inmates as of mid-year 2006, a total of 2,245,189 adults were incarcerated in the US on June 30, 2006 [5].

### Number of Individuals Passing through Correctional Facilities, 2006

From the 2006 Annual Survey of Jails data, using the method of Sabol and Minton [16], we calculated that there were 12.8 million admissions to jails in 2006. Data from the sources listed in Table 1 gave a mean value for the number of incarcerations per individual of 1.4. Dividing the total number of admissions by the number of jailings per individual yields 9 million unique persons admitted to jails in 2006 ([a+d] in Figure S1). The number of releasees was found by taking the difference between the mid-year US jail population in 2006 (766,010) and 2005 (747,529). From mid-year 2005 to mid-year 2006, the jail population grew by 18,481, meaning there were that many fewer releases than admissions. Therefore, 8,981,519 unique persons were released from jail to the community or prison in the year preceding June 30, 2006 ([b+c+e+g+h] in Figure S1).

Records from 2003 NCRP data on all prison admissions from 34 states showed that 354,120 unique persons were admitted in these 34 states. This figure represents 71.95% of the number of prison admissions reported by BJS (for concordant states, when outliers California and North Carolina are ignored; see Methods). According to BJS, in 2003 there were 96,416 prison admissions in the 16 states not covered by NCRP. Reducing the total number of admissions by the same factor (71.95%) yielded 69,376 unique admittees. Added to the states already covered by NCRP, we arrived at 423,496 unique admittees to state prison in 2003. Comparison of 2002 and 2003 admission data from NCRP showed very little variation between years, leading us to conclude that the data were stable enough for short-term extrapolation. In order to extrapolate to 2006, we examined the trend of state prison admission data between 2000 and 2006 as reported by BJS. Year-to-year changes varied by factors ranging from 1.0157 to 1.0346 (average 1.0259). We used this average growth rate to project a 2006 estimate from our 2003 estimate to arrive at 457,261 unique admittees to state prisons in 2006. Adding the 57,495 admissions to federal prisons to this figure yields an estimate of 514,756 unique admissions to all US prisons in 2006.

Of the 514,756 unique admissions, 70.84% (364,653) of individuals sentenced to prison were detained pretrial ([e] in Figure S1). The difference in these two figures (150,103 individuals) was admitted directly from the street. The number of jail detainees released to the community should therefore be 364,653 less than the number of total jail releasees, or 8,616,866 individuals ([b+c+g+h] in Figure S1).

The same methodology was applied to estimate 414,731 unique releasees from state prison in 2003. To extrapolate to 2006, we examined the trend of state prison release data between 2000 and 2006 as reported by BJS. With the exception of the year 2002, which saw a 1% drop in releases, year-to-year growth varied by factors ranging from 1.0219 to 1.0398 (average 1.0270). Applying this growth rate to the 2003 estimate from NCRP data yielded 449,240 unique releasees from state prisons in 2006. Adding to this the 47,920 federal releasees, there were 497,160 unique prison releasees ([j+k] in Figure S1).

To derive the number of people in a CF at any point in 2006, we added the number of unique jail admissions, the number of prison admissions from the street, and the stock prison population at the beginning of 2006 (the 1,525,924 persons in prisons the last day of 2005). This calculation yielded 10,676,027 individuals. In order to calculate the number of persons released to the community, we summed the unique jail and prison releasees to yield 9,114,026 individuals (Tables 2 and 3).

**Table 2:** Unique Persons Ever in a Correctional Facility during 2006.

	Unique Persons	Percentage
Admittees to jail	9,000,000	84.3%
Prisoners admitted from street	150,103	1.4%
Stock population in prison, end of 2005	1,525,924	14.3%
Number Inside at Some Point	10,676,027	100%

**Table 3:** Releasees from All Correctional Facilities in 2006.

	Unique Persons	Percentage
Released from jail to street	8,616,866	95%
Unique releasees from prison	497,160	5%
Total releasees	9,114,026	100%

To examine the sex and race/ethnicity composition of those ever in and those released from CFs, we weighted the population by the sex and race/ethnicity makeup of the constituent parts. For the total number of persons who were ever in a CF in 2006, about 85% were jail inmates. Releasees were composed of about 95% jail detainees and 5% prisoners. Estimates of the correctional population broken down by sex and race/ethnicity are shown in Tables 4 and 5.

**Table 4:** Sex and Race/Ethnicity Stratification of Unique Persons Ever in a Correctional Facility during 2006.

Men		Women		Total
Total	9,519,692	Total	1,156,335	10,676,027
White	3,309,805	White	502,776	
Black	3,866,617	Black	420,671	
Hispanic	1,824,222	Hispanic	159,518	
Other*	519,048	Other*	73,370	

* Refers to persons specifying mixed or other race/ethnicity, or not specifying any race or ethnicity.

**Table 5:** Sex and Race/Ethnicity Stratification of Releasees from All Correctional Facilities in 2006.

Men		Women		Total
Total	8,081,581	Total	1,032,922	9,114,026
White	2,821,854	White	448,539	
Black	3,276,175	Black	377,964	
Hispanic	1,545,270	Hispanic	141,139	
Other*	437,861	Other*	65,225	

* Refers to persons specifying mixed or other race/ethnicity, or not specifying any race or ethnicity.

For comparative purposes, we recalculated our 1997 estimates of releasees downward from 7.8 million, using the same correction coefficients. We arrived at 384,009 unique persons admitted to state and federal prisons in 1997. Applied to the 7.2 million jail releasees used in the initial paper, we obtained a revised estimate of 6.93 million jail releasees. NCRP data on prison releases in 1997 yielded an estimate of 368,263 unique releasees from state and federal prisons. Thus, the revised estimate of unique releasees from jails and prisons in 1997 is 7.30 million. This revised estimate for the denominator changes estimates of the epidemic borne by the correctional population only moderately (Table 6).

**Table 6:** Revised 1997 Estimate of Share of HIV/AIDS among Correctional Facility Releasees.

	Previous Estimate for 1997	Revised Estimate for 1997
AIDS	15.7%	14.8%
HIV/AIDS	20.1–26.2%	19.0–24.6%
HIV/AIDS (midpoint)	23.2%	21.8%

### HIV/AIDS: Period Prevalence among Inmates and Proportion of the US Epidemic

Based on the varying testing practices among prison systems, the BJS, in *HIV in Prisons, 2006*, reported prison HIV/AIDS prevalence of 1.7% and an AIDS prevalence of 0.5% [4]. Because some of these states do not routinely test for HIV, this estimate may be inaccurate, but the bias could be in either a positive or negative direction.

In a nationally representative survey of jail inmates' medical problems conducted in 2002, 1.3% of jail detainees reported themselves to be HIV positive [24]. Of those surveyed however, 37.1% had never been tested [19], [23]. MacGowan has shown that approximately 0.8% of all jail inmates with unknown HIV status are positive [10]. If 0.8% of the 37.1% of untested inmates are also positive, it raises the overall jail HIV prevalence estimate to 1.6%, which is almost as high as the estimate for prisons. As a previous paper noted, jail and prison populations have similarities in demographics related to HIV risk.[1] We applied an HIV prevalence of 1.7% to both jail and prison populations.

Tables 7 and 8 show the estimated number of persons infected, by race and sex. The HIV prevalence data by race and sex, from the *2004 Survey of State Inmates* (available on the NCRP website), were multiplied by the number of persons in the demographic category to give estimated crude estimates of the number of persons infected, which are shown in Tables 7 and 8. If none of the HIV-infected men ever in a CF classified as “other” were black, then 73,466 black men would be infected; if all of the HIV-infected male prisoners classified as “other” were black, then 92,591 of black men would be infected. Possible ranges for the numbers in other demographic groups among those ever in a CF are shown in Table 7.

**Table 7:** HIV/AIDS among Populations Ever in a Correctional Facility (CF) during 2006.

Demographic Group	Total CF Population	HIV Infections in Total Population Ever in CF During the Year	HIV Infections in Total US Population	HIV Borne by Total Population Ever in CF During the Year	AIDS Borne by Total CF Population/US Total
Total	10,676,027	187,274	1,106,400	16.9%	53,380/447,720 (11.9%)
Total Men	9,519,692	161,835	828,000	19.5%	
White	3,309,805	36,408–55,533	328,000	11.1%–16.9%	
Black	3,866,617	73,466–92,591	332,000	22.1%–27.9%	
Hispanic	1,824,222	32,836–51,956	151,000	21.7%–33.4%	
Other	519,048	*			
Total Women	1156335	25,439	278,400	9.1%	
White	502,776	2,514–6,829	55,100	4.6%–12.4%	
Black	420,671	14,303–18,618	177,200	8.1%–10.5%	
Hispanic	159,518	4,307–8,622	41,600	10.4%–20.7%	
Other	73,370	*			

**Table 8:** HIV/AIDS among Releasees from Correctional Facilities in 2006.

Demographic Group	Releasees	HIV Infections in Releasees	HIV Infections in Total US Population	HIV Borne by Releasees	AIDS Borne by Releasees/US Total
Total	9,114,026	154,938	1,106,400	14.0%	45,570/447,720 (10.2%)
Total Men	8,081,581	137,380	828,000	16.6%	
White	2,821,854	31,040–47,381	328,000	9.5%–14.4%	
Black	3,276,175	62,247–78,525	332,000	18.7%–23.7%	
Hispanic	1,545,270	27,815–44,093	151,000	18.4%–29.2%	
Other	437,861	*			
Total Women	1,032,922	22,723	278,400	8.2%	
White	448,539	2,243–6,061	55,100	4.1%–11.0%	
Black	377,964	12,851–16,669	177,200	7.3%–9.4%	
Hispanic	141,139	3,811–7,629	41,600	9.2%–18.3%	
Other	65,225	*			

The revised CDC methodology estimated that 1,106,400 persons were living with HIV (range: 1,056,400–1,156,400) in the US (50 states and the District of Columbia) in 2006 [21]. Based on our estimates of 10,642,946 persons ever in a CF in 2006 and a prevalence of 1.7%, 16.9% of all Americans with HIV/AIDS were in a CF at some point in 2006. If none of the HIV-infected male prisoners classified as “other” were black, then 22.1% of black men living with HIV in the US would pass through a CF in 2006; if all of the HIV-infected male prisoners classified as “other” were black, then 27.9% of black men living with HIV would pass through a US facility in the same year. Possible ranges for the percentages for other demographic groups are shown in Table 7.

The CDC estimate for the number of persons living with AIDS in the 50 states and the District of Columbia in 2006 was 447,720 [22]. If the prevalence of AIDS in correctional populations is 0.5%, then 11.9% of all individuals with AIDS were in a CF at some point in 2006.

Table 8 shows similar calculations for the proportion of the HIV epidemic borne by the 9,114,026 releasees from CFs in 2006. Of all Americans with HIV/AIDS, 154,938 (14.0%) were released from a CF that year. This represents a 29.3% decline from 1997 (Table 9). The proportion of the US black male HIV epidemic represented by black men released from CFs in 2006 is likely between 18.7% and 23.7% (Table 8). Of all Americans with AIDS, 45,570 (10.2%) were released from a CF at some point that year (Table 8). This represents a 31.1% decline from 1997 (Table 9).

**Table 9:** Decline in Share of HIV and AIDS Borne by Releasees without a Substantial Change in Numbers Affected.

Variable	Revised Estimate for 1997	Estimate for 2006	Percent Change
Share of HIV/AIDS	19.8% [95% CI: 17.0–24.5%]	14.0%	29.3% decline
Share of AIDS	14.8%	10.2%	31.1% decline
Number of persons with HIV among releasees	153,300	154,938	1.1% increase

### Sensitivity Analysis

We assessed the stability of our estimate of the proportion of HIV/AIDS borne by releasees from CFs. Treating all 12.8 million jail releasees and 713,473 prison releasees reported by the BJS as unique persons and using the lower bound of the CDC estimate of HIV/AIDS prevalence in the US, the proportion of HIV/AIDS borne by releasees is 20.6%, which is lower than the midpoint of our revised estimate for 1997 and near the lower limit of the 1997 range. Given the uncertainty of the earlier estimate, we can conclude that even if our estimates of unique releasees were grossly inaccurate, the proportion would not have shifted substantially from 1997 levels. To calculate a lower bound, we assumed that the average number of incarcerations per jail inmate was 1.5 (the highest return rate found in sources of data for Table 1). Using this return rate yielded an estimated proportion of HIV/AIDS borne by releases from CFs of 12.7%. The range of the estimate was therefore 12.7% to 20.6%.

If 12.8 million jail admissions in 2006 represented individuals who each were incarcerated 1.5 times rather than 1.4 times per year, approximately 500,000 fewer persons were admitted to jail. This adjustment would not change the number of persons who were released from jail to go to prison or who were released from prison; therefore, the absolute number of releasees would decline by 500,000. Assuming an HIV prevalence of 1.7%, this adjustment would mean that 8,500 fewer HIV-infected persons (4.5% fewer than previously estimated) were released from correctional facilities in 2006.

## Discussion

We suggest four factors that could explain the diminishing proportion of HIV/AIDS borne by the populations moving through CFs; these four factors could contribute either alone or in combination. First, with increased life expectancy for persons with HIV with the advent of better therapeutics, infected persons are aging out of the crime-prone years, generally considered to be between the ages of 15 and 24. Second, while a person may have been infected in his or her crime-prone years, with HAART, prison AIDS mortality has fallen [20], and that person is more likely to survive incarceration, be released, and stay out. Third, the past decade has seen a decline in the number and proportion of HIV/AIDS cases among injection drug users [21], [22], probably due to interventions to reduce the harm associated with parenteral drug use.

Finally, considerable effort has been made by prison systems, and some jails, to enhance discharge planning for HIV-infected persons [24]. The effects of these programs are difficult to assess. A controlled but non-randomized trial has been conducted in North Carolina [25], [26]. Preliminary results show a non-significant trend in the efficacy of discharge planning, but final results have yet to be published as of submission of this manuscript. Observational studies seem to show that those CFs with adequate discharge planning do better than those without [27]. A program in Rhode Island compared the recidivism of HIV-infected women to historical controls and found a significant difference in return rates [28]. Enhanced discharge planning of HIV-infected inmates beyond that usually received by prisoners may be contributing to lower recidivism rates in the HIV-infected population in Rhode Island.

One limitation of this study was the need to make numerous assumptions about the proportion of persons who would move from a jail to a prison and the number of releasees who would return to the same type of facility in the space of 1 y. The sensitivity analysis shows that even with faulty estimates, the general trend is probably accurate. We emphasize the unlikelihood of all jail and prison releasees representing unique persons. A second limitation is that our recidivism data were based on rates of return to the same institution. The assumption that recidivists would be reincarcerated in the identical jurisdiction is not far-fetched, given that many releasees would be on probation or parole, which often stipulates residency in the same jurisdiction. Third, HIV/AIDS prevalence in prisons and jails was largely derived from self-reported data, and an estimate of the prevalence in untested individuals was applied. We believe this would bias our estimate of inmates with HIV/AIDS slightly downwards, if at all.

Because certain groups at high risk for HIV/AIDS come together in correctional facilities, seroprevalence was high early in the epidemic; 16.2% of men and 25.1% of women tested for HIV in 1989 at the New York City jail on Rikers Island were found to be HIV-positive [29]. The decline in HIV prevalence among correctional populations has been offset by the growing number of inmates. Although the proportional share of HIV/AIDS borne by those passing through CFs has declined since 1997, the total number of HIV infected persons who are in this flow has remained steady at roughly 150,000 individuals, an estimate that is only marginally perturbed by an assumption that each detainee is incarcerated 1.5 rather than 1.4 times per year.

As the HIV epidemic has matured, the share borne by releasees has decreased, but the total number of persons with HIV released from CFs is unchanged. This steady size of the target population leads us to conclude that CFs still represent a rich focus for public health interventions. Interventions in CFs may have the greatest impact on the HIV epidemic among minority men, given the disproportionate incarceration rates in the US criminal justice system. The proportion of minorities diagnosed with HIV late in the course of disease, less than 12 mo before a diagnosis of AIDS, continues to lag behind whites [30], so reaching minority populations is a public health priority. Jail and prison inmates represent a captive, and still very important, audience for HIV testing, counseling, and prevention messages. After diagnosis, enabling HIV-infected releasees to link to community care is of utmost importance. Because virtually all persons entering CFs return to the community, effective interventions benefit not only CF populations but also the communities to which releasees return.

## Supporting Information

Figure S1 Populations Flowing through Correctional Facilities
(1.61 MB TIF)
