# Supplementary figures and images for: HIV/AIDS among Inmates of and Releasees from US Correctional Facilities, 2006: Declining Share of Epidemic but Persistent Public Health Opportunity
Source: PLoS One. 2009 Nov 11;4(11):e7558. doi: 10.1371/journal.pone.0007558 (PMC2771281; doi:10.1371/journal.pone.0007558)

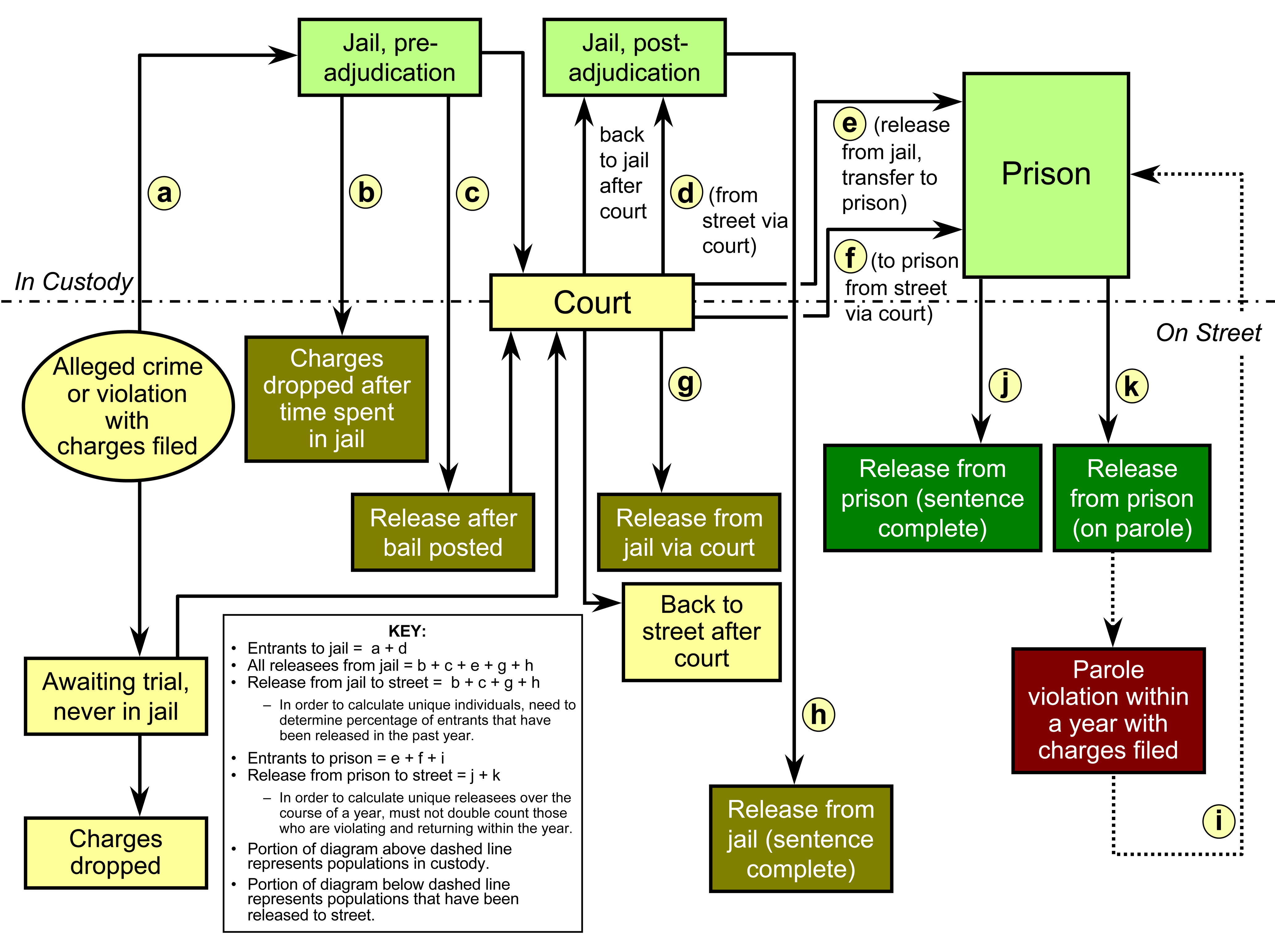

Supplement: Figure S1 — Populations Flowing through Correctional Facilities (1.61 MB TIF) [file pone.0007558.s001.tif]
